# Prediction of Poor Responders to Neoadjuvant Chemotherapy in Patients with Osteosarcoma: Additive Value of Diffusion-Weighted MRI including Volumetric Analysis to Standard MRI at 3T

**DOI:** 10.1371/journal.pone.0229983

**Published:** 2020-03-10

**Authors:** Seul Ki Lee, Won-Hee Jee, Chan Kwon Jung, Soo Ah Im, Nack-Gyun Chung, Yang-Guk Chung

**Affiliations:** 1 Department of Radiology, Seoul St. Mary’s Hospital, College of Medicine, The Catholic University of Korea, Seocho-gu, Seoul, Republic of Korea; 2 Department of Pathology, Seoul St. Mary’s Hospital, College of Medicine, The Catholic University of Korea, Seocho-gu, Seoul, Republic of Korea; 3 Department of Pediatrics, Seoul St. Mary’s Hospital, College of Medicine, The Catholic University of Korea, Seocho-gu, Seoul, Republic of Korea; 4 Department of Orthopedic Surgery, Seoul St. Mary’s Hospital, College of Medicine, The Catholic University of Korea, Seocho-gu, Seoul, Republic of Korea; Washington University in St. Louis School of Medicine, UNITED STATES

## Abstract

**Objective:**

To evaluate the added value of diffusion weighted image (DWI) including volumetric analysis to standard magnetic resonance imaging (MRI) for predicting poor responders to neoadjuvant chemotherapy in patients with osteosarcoma at 3-Tesla.

**Methods:**

3-Tesla Standard MRI and DWI in 17 patients were reviewed by two independent readers. Standard MRI was reviewed using a five-level-confidence score. Two-dimensional (2D) apparent diffusion coefficient (ADC)_mean_ and 2D ADC_minimum_ were measured from a single-section region of interest. An ADC histogram derived from whole-tumor volume was generated including 3D ADC_mean_, 3D ADC_skewness_, and 3D ADC_kurtosis_. The Mann-Whitney-*U* test, receiver operating characteristic curve with area under the curve (AUC) analysis, and multivariate logistic regression analysis were performed.

**Results:**

There were 13 poor responders and 4 good responders. Statistical differences were found in posttreatment and percent change of both 2D ADC_mean_ and 2D ADC_minimum_, posttreatment 3D ADC_mean_, and posttreatment 3D ADC_skewness_ between two groups. The best predictors of poor responders were posttreatment 2D ADC_mean_ and posttreatment 3D ADC_skewness_. Sensitivity and specificity of the 1^st^ model (standard MRI alone), 2^nd^ model (standard MRI+posttreatment 2D ADC_mean_), and 3^rd^ model (standard MRI+posttreatment 2D ADC_mean_+posttreatment 3D ADC_skewness_) were 85% and 25%, 85% and 75%, and 85% and 100% for reader 1 and 77% and 25%, 77% and 50%, and 85% and 100% for reader 2, respectively. The AUC of the 1^st^, 2^nd^, and 3^rd^ models were 0.548, 0.798, and 0.923 for reader 1 and 0.510, 0.635, and 0.923 for reader 2, respectively.

**Conclusion:**

The addition of DWI including volumetric analysis to standard MRI improves the diagnostic accuracy for predicting poor responders to neoadjuvant chemotherapy in patients with osteosarcoma at 3-Tesla.

## Introduction

Nonmetastatic osteosarcoma is currently treated with neoadjuvant chemotherapy before surgery [1, 2]. The histologic response after resection reflects the efficacy of neoadjuvant chemotherapy [3]. If the treatment response could be assessed earlier, this information may help avoid ineffective chemotherapy and determine surgical timing [4, 5].

Magnetic resonance imaging (MRI) and fluorine-18 fluorodeoxyglucose (^18^F FDG) combined positron emission tomography (PET)/computed tomography (CT) using maximum standardized uptake value (SUV_max_) have been used to assess osteosarcoma during neoadjuvant chemotherapy. ^18^F FDG PET/CT assesses the glucose metabolism and calculates the metabolic activity of tumor by SUV [6]. Change of SUV after neoadjuvant chemotherapy in osteosarcoma has been demonstrated to be useful in predicting treatment response [7–9]. However, the delineation of tumor margins on ^18^F FDG PET/CT is difficult and monitoring responses is problematic when the uptake is increased by inflammation or reactive fibrosis [6, 8]. Viable tumors showed strong enhancement without a decrease in tumor size in several previous studies on standard MRI [10–12]. However, standard MRI has limited ability to assess treatment responses because treated lesions sometimes show remnant contrast enhancement and often increase in size despite pathological response.

Posttreatment changes, such as tumor necrosis or a reduction in cell density, cause expansion of the extracellular diffusion space [13]. Diffusion-weighted imaging (DWI) can measure these changes as an increase in apparent diffusion coefficient (ADC) after neoadjuvant chemotherapy. For the osteosarcoma, many studies have assessed the treatment response to neoadjuvant chemotherapy using ADC values; however, the results of previous reports are inconsistent [6, 10, 14–17]. This inconsistency may be attributed to the several differences in techniques of DWI sequences among studies and/or region of interest (ROI) measurement to reflect the whole tumor heterogeneity in a single section. The value of the whole-tumor volume analysis of the ADC map to evaluate the treatment response of osteosarcoma has not been fully demonstrated in the literature, which may complement these limitations [18–20].

Therefore, we hypothesized that DWI including a volumetric analysis may improve the diagnostic performance for predicting poor responders to neoadjuvant chemotherapy in patients with osteosarcoma at 3T.

## Materials and methods

### Patients

**Fig 1:**
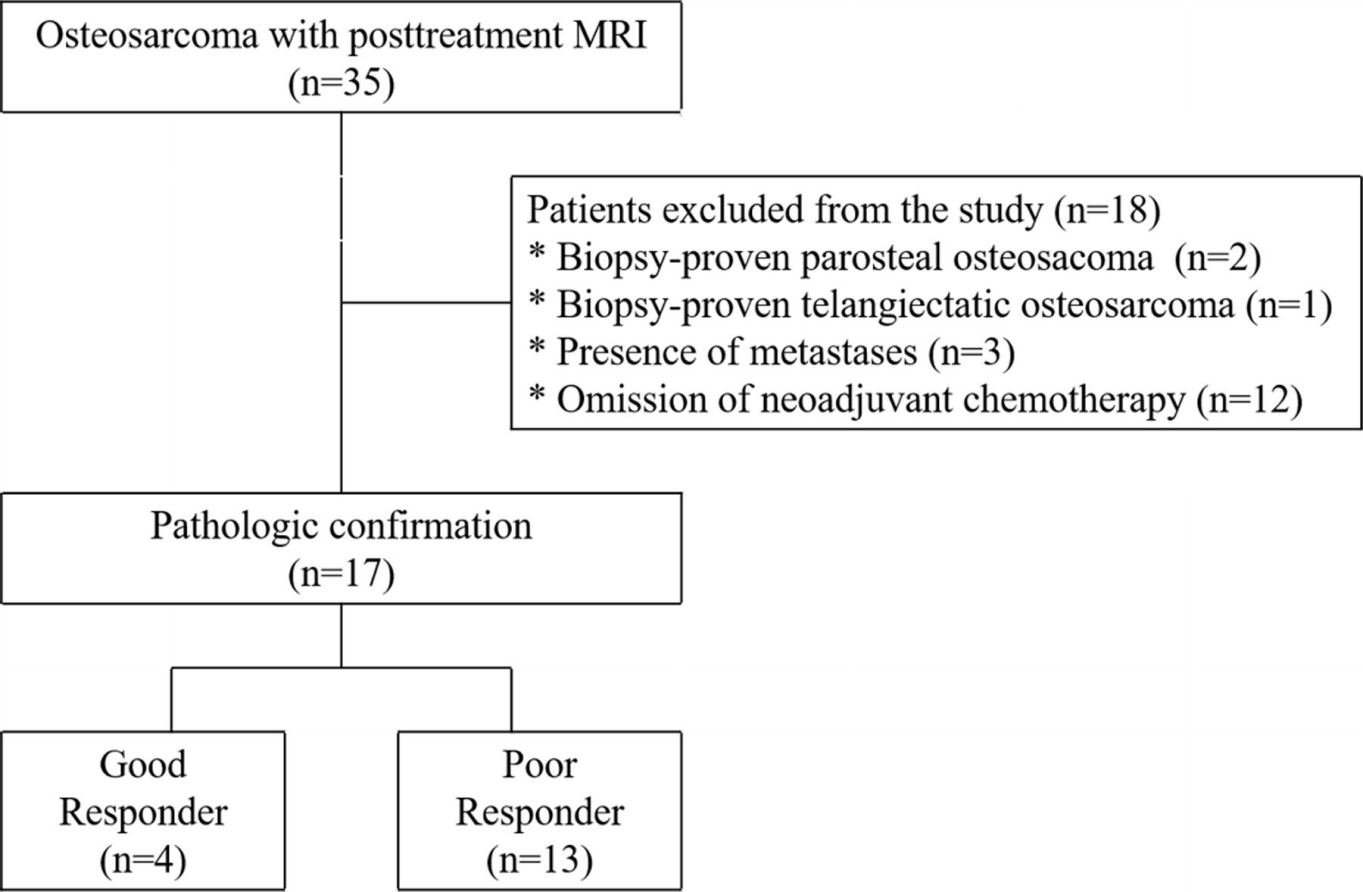
Flow diagram of the study. MRI = magnetic resonance imaging.

The Seoul St. Mary’s Hospital Institutional Review Board approved this retrospective study and waived the need for informed consent. Thirty-five consecutive patients with osteosarcoma were admitted between March 2009 and May 2017. The inclusion criteria were: (a) conventional osteosarcoma, (b) no identified metastases, (c) 3T MRI including DWI after neoadjuvant chemotherapy, (d) and histologic specimen analysis after surgery. Eighteen patients were excluded for the following reasons: parosteal osteosarcoma (n = 2), telangiectatic osteosarcoma (n = 1), metastatic disease (n = 3), and omission of neoadjuvant chemotherapy (n = 12). Finally, 17 patients (mean age, 17 years [range, 10–53 years]; 13 males) were included (Fig 1). Neoadjuvant chemotherapy was decided using the Children’s Cancer Group (CCG)-7921 regimen A in 12 patients [21]. Four patients did not receive Methotrexate (MTX) at secondary cycle by the monitoring of plasma concentrations. One patient received only one cycle of CCG-7921 regimen A and one cycle of ifosfamide and etoposide because of progressively increasing size. The median interval was 10 days (range, 1–37 days) between neoadjuvant chemotherapy and posttreatment MRI, 109 days (range, 78–166 days) between pretreatment and posttreatment MRI, and 4 days (range, 1–25 days) between posttreatment MRI and surgery. Tumors were located in the femur (n = 9), tibia (n = 4), humerus (n = 3), and scapula (n = 1). Histological subtypes were osteoblastic osteosarcoma (n = 13), fibroblastic osteosarcoma (n = 3), and chondroblastic osteosarcoma (n = 1).

### MRI protocols

All 17 patients underwent posttreatment MRI including DWI. Among them, 11 had pretreatment MRI including DWI, while the other 6 patients had only pretreatment standard MRI with no DWI. MRI was performed using a 3T scanner (MAGNETOM Verio; Siemens Healthineers, Erlangen, Germany) with various coils depending on the anatomic region. MRI protocols included longitudinal fat-suppressed (FS) turbo spin-echo (TSE) T2-weighted imaging (T2WI), axial TSE T1-weighted imaging (T1WI), axial TSE T2WI with and without FS, and longitudinal and axial FS contrast-enhanced TSE T1WI. T1WI (TR/TE = 680–870 msec/11–21 msec, turbo factor = 3, number of excitations = 1) and T2WI (TR/TE = 4000–5600 msec/63–83 msec, turbo factor = 13, number of excitation = 1) was obtained with 3–5-mm slice thickness, no interslice gap, an 80–220-mm field of view (FOV), and 512 × 256 matrix size.

DWI was acquired in the axial plane using a single-shot echoplanar imaging sequence. The DWI parameters were as follows: TR/TE, 5000–8700 msec/71–85 msec; FOV, 80–220 mm; slice thickness, 3–5 mm; no interslice gap; matrix size, 80 × 56–128 × 108; EPI factor, 56; and number of excitations, 3–5. Diffusion-sensitizing gradients were applied sequentially in 3 orthogonal directions with four *b* values (0, 300, 800, and 1400 sec/mm^2^) in 14 patients and intravoxel incoherent motion DWI with 9 *b* values (0, 25, 50, 75, 100, 200, 300, 500, and 800 sec/mm^2^) in the other 14 patients. Pixel-based ADC maps were created based on monoexponential fitting using common *b* values of 0 and 800 sec/mm^2^ using commercial software and a workstation (Leonardo MR Workplace; Siemens Healthineers, Erlangen, Germany).

### MRI analysis

Standard MRI analysis for treatment response was performed independently by 2 musculoskeletal radiologists (W.H.J, S.K.L, with 17 and 2 years of experience in musculoskeletal radiology) who were blinded to the patients’ clinical histories, MRI reports, surgical findings, and histopathological results. Standard MRI of treatment responses were assessed using a 5-level confidence score: 0, definite good response; 1, probable good response; 2, equivocal; 3, probable poor response; and 4, definite poor response. In the review of standard MRI, pre- and posttreatment images were available for all patients. Therefore, both images were analyzed simultaneously. According to Lang et al. [22], there was no significant difference on T2WI between viable and necrotic tumor tissue because the T2 relaxation times were similar. Therefore, we used contrast-enhanced T1WI to evaluate the viable tumor. When there was an intense enhanced portion at most of area of tumor without interval decrease in extent of enhanced area and size reduction on a posttreatment image, it was considered a definite poor response (score 4) on standard MRI [10–12]. If most of area of tumor was enhanced, despite interval decrease in extent of enhanced area, it was considered a probable poor response (score 3). When the heterogeneous enhancement remained on tumor, despite interval decrease in extent of enhanced area, it was considered an equivocal case (score 3). If most of area of tumor was not enhanced on the posttreatment image, it was considered a probable good response (score 1). When there was little enhancement with size reduction on the posttreament image, it was considered a definite good response (score 0).

For the single-section ROI of the DWI analysis, the same two readers independently reviewed the DWI with display of standard MRI for the correlation of the solid portion in a picture archiving and communication system. If present, pretreatment DWI was also referenced and analyzed. Two readers independently drew two freehand ROI on a single representative section: 1) mean ADC obtained from the single-section ROI (2D ADC_mean_)–ROI that contained the largest area of the tumor except for the peripheral most portions to avoid partial-volume effects. The representative axial slice was carefully selected with reference of standard MRI in order to avoid any necrosis, cystic change, hemorrhage, and sclerosis that might affect the ADC values; and 2) minimum ADC obtained from the single-section ROI (2D ADC_minimum_)–ROI located in the lowest signal intensity (SI) within the solid portion of the tumor on the ADC map that presented as a hyperintense SI on DWI with a *b* value of 800 sec/mm^2^. To select the lowest ADC value, small ROI (minimum area, 0.5 cm^2^) were drawn 3–5 times and the minimum was recorded [23].

For the whole-tumor volume analysis, the other reader (S.A.I) who was blinded to the patients’ clinical histories, MRI reports, surgical findings, and histopathological results reviewed the DWI using the MR OncoTreat software (provided by Siemens Healthineers, Erlangen, Germany). A freehand ROI was drawn along the border of the tumor on DWI with a *b* value of 800 sec/mm^2^ on each tumor-containing slice including the solid portion, necrosis, cystic change, hemorrhage, and sclerosis. And then, the software automatically computed the ADC histograms. The mean ADC obtained from the ADC histogram of whole-tumor volume (3D ADC_mean_) was recorded. Skewness and kurtosis were also generated from the ADC histogram of the whole-tumor volume, which reflected the shape of the histogram. Skewness obtained from the whole-tumor volume (3D ADC_skewness_) represents the asymmetry of the ADC value distribution around the mean. A negative skewness indicates that most of the data are concentrated on the right (left-skewed curve). Kurtosis obtained from the whole-tumor volume (3D ADC_kurtosis_) represents the peak and size of the data distribution. A normal distribution shows a skewness of 0 and kurtosis of 3 [24, 25].

The percent change in parameters was calculated if available. The formula used was as follows: Percent change = [(Parameterposttreatment—Parameter_pretreatment_)/Parameter_pretreatment_] × 100.

### Pathological analysis

One pathologist (C.K.J) assessed degree of tumor necrosis using the 4-grade system of Huvos [3, 4]. The resected tumor was fixed in a 10% formaldehyde solution and a representative complete central slab of the specimen was entirely embedded in a grid-like manner. The representative tissue slab was selected and assessed macroscopically, which should reflect the response level of the whole tumor [26]. Based on the histologic analysis, a good responder was defined as >90% tumor necrosis.

### Statistical analysis

Interobserver agreement for the single-section measurement was evaluated by the Bland-Altman method [27], while the comparison of data between two groups was performed using Mann-Whitney *U*-test. Diagnostic performances were analyzed using receiver operating characteristic (ROC) curve with areas under the curve (AUC). Sensitivities and specificities were calculated. To examine independent predictive parameters for predicting poor responders, multivariate logistic regression analysis was used. Values of *P* < 0.05 were considered statistically significant. All statistical analyses were performed using SPSS Statistics (IBM Corporation, Chicago, IL, USA) and MedCalc (MedCalc, Mariakerke, Belgium).

## Results

There were four good responders (mean age, 17 years [range, 15–20 years]; 3 males) and 13 poor responders (mean age, 16 years [range, 10–53 years]; 10 males) (*P* > 0.05).

### Standard MRI analysis of treatment response

Standard MRI after neoadjuvant chemotherapy showed significant non-enhancing portions within tumors (score 1) in three patients for reader 1 and in 4 patients for reader 2. Among them, only 1 patient was a good responder on pathological analysis for both readers. Standard MRI after neoadjuvant chemotherapy showed significant enhancement within the tumors (score 3 or 4) of 11 patients for both readers. Among them, 10 patients were identified as poor responders on pathological analysis for both readers and only 1 patient was a good responder on pathological analysis for both readers. The standard MRI showed equivocal (score 2) results for three patients for reader 1 and for 2 patients for reader 2. Two of each were good responders on pathological analysis. Table 1 summarizes the result of a 5-level confidence score for treatment response on standard MRI for both readers.

**Table 1:** Results of standard MRI analysis and diagnostic performance for treatment response of osteosarcoma.

5-confidence level	Reader 1					Reader 2				
	Poor responder (n = 13)			Good responder (n = 4)		Poor responder (n = 13)			Good responder (n = 4)	
Score 0, definitely good response	0			0		0			0	
Score 1, probably good response	2			1		3			1	
Score 2, equivocal	1			2		0			2	
Score 3, probably poor response	9			1		10			0	
Score 4, definitely poor response	1			0		0			1	
Cutoff ≥ Score 2 suggesting poor responder	Sensitivity	Specificity	AUC	Sensitivity	Specificity	AUC
	85%	25%	0.740	77%	25%	0.606

AUC, areas under the curve.

### DWI and ADC map analysis of treatment response

**Fig 2:**
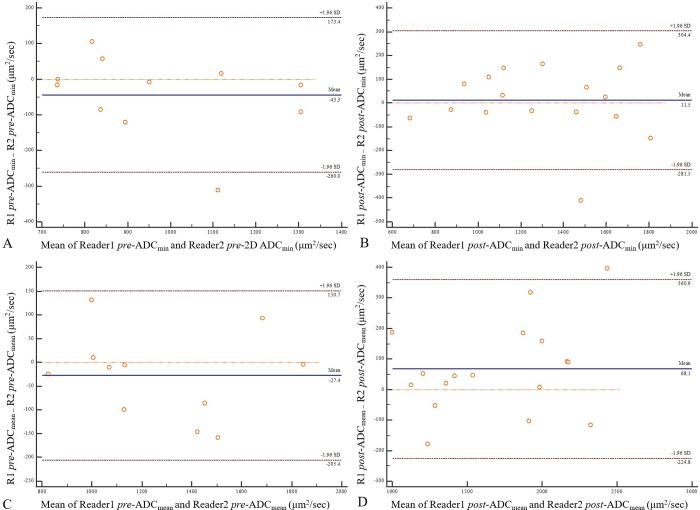
Bland-Altman plots of ADC measurements derived from single-section ROIs between two readers. (A) pretreatment 2D ADC_minimum_, (B) posttreatment 2D ADC_minimum_, (C) pretreatment 2D ADC_mean_, and (D) posttreatment 2D ADC_mean_. The unit of ADC is μm^2^/sec. pre- = pretreatment; post- = posttreatment; ADC_min_ = ADC_minimum_.

A pretreatment DWI was lacking for 6 patients. For reader 1, the posttreatment 2D ADC_minimum_ and posttreatment 2D ADC_mean_ were significantly lower in poor responders than in good responders (*P* = 0.024 and *P* = 0.017, respectively). In 11 cases with available pretreatment DWI, significantly different percent changes between good and poor responders were found in 2D ADC_mean_, 80.0% vs. 9.5% for reader 1 and 2D ADC_minimum_, 71.9% vs. 19.0% for reader 2 (*P* = 0.034 for both). Comparisons of pretreatment, posttreatment, and percent change of ADC values derived from single-section ROI (2D ADC) between the two groups are summarized in Table 2. Interobserver agreement for 2D ADC_minimum_ showed that the mean difference (bias) and the 95% confidence interval (CI) of the mean difference (limits of agreement) were -43.27 μm^2^/sec (-259.96, 173.42) at pretreatment and 11.47 μm^2^/sec (-281.50, 304.44) at posttreatment. Interobserver agreement of posttreatment 2D ADC_minimum_ was superior to that of pretreatment 2D ADC_minimum_ (Fig 2). For 2D ADC_mean_, -27.36 μm^2^/sec (-205.42, 150.69) at pretreatment and 68.05 μm^2^/sec (-224.79, 360.90) at posttreatment were identified. Interobserver agreement of pretreatment 2D ADC_mean_ was superior to that of posttreatment 2D ADC_mean_ (Fig 2).

**Table 2:** Comparison of 2D ADC measurement for treatment response of osteosarcoma.

Parameters	Poor responder	Good responder	*P*
Pretreatment 2D ADC_minimum_	n = 9	n = 2	
Reader 1	870 [795;956]	999 [870;1127]	0.555
Reader 2	955 [813;1268]	939 [765;1112]	0.813
Pretreatment 2D ADC_mean_	n = 9	n = 2	
Reader 1	1130 [1065;1426]	1180 [1011;1349]	0.478
Reader 2	1179 [1076;1585]	1248 [1001;1495]	0.637
Posttreatment 2D ADC_minimum_	n = 13	n = 4	
Reader 1	1195 [1017;1384]	1613 [1575;1751]	0.024*
Reader 2	1099 [998;1481]	1610 [1531;1656]	0.089
Posttreatment 2D ADC_mean_	n = 13	n = 4	
Reader 1	1439 [1232;1968]	2151 [2081;2426]	0.017*
Reader 2	1395 [1311;1964]	2025 [1843;2182]	0.089
Percent change 2D ADC_minimum_	n = 9	n = 2	
Reader 1	30 [17;38]	60 [44;77]	0.099
Reader 2	19 [-3;21]	72 [51;93]	0.034*
Percent change 2D ADC_mean_	n = 9	n = 2	
Reader 1	10 [8;28]	80 [54;106]	0.034*
Reader 2	9 [-2;23]	55 [18;92]	0.239

2D ADC, apparent diffusion coefficient values derived from single-section regions of interest.

*indicates statistical significance.

The whole-tumor volume analysis revealed significantly lower posttreatment 3D ADC_mean_ in poor responders than in good responders (*P* = 0.042). Poor responders demonstrated significantly higher posttreatment 3D ADC_skewness_ than good responders (*P* = 0.017). However, there was no statistical significance in 3D ADC_kurtosis_ (*P* > 0.05). Comparisons of pretreatment, posttreatment, and percent change of ADC values derived from whole-tumor volume (3D ADC) between the two groups are summarized in Table 3.

**Table 3:** Comparison of 3D ADC measurement for treatment response of osteosarcoma.

Parameters	Poor responder	Good responder	*P*
	n = 9	n = 2	
Pretreatment 3D ADC_mean_	1472.7 [1128.1;1784.3]	1228.4 [943.2;1513.5]	0.480
Pretreatment 3D ADC_skewness_	0.3 [-0.5; 0.7]	0.4 [-0.7; 1.5]	0.814
Pretreatment 3D ADC_kurtosis_	4.7 [4.0; 5.8]	5.4 [4.0; 6.8]	0.637
	n = 13	n = 4	
Posttreatment 3D ADC_mean_	1574.3 [1309.6;1864.2]	2053.6 [1967.2;2224.6]	0.042*
Posttreatment 3D ADC_skewness_	-0.0 [-0.4; 0.4]	-0.9 [-1.2;-0.8]	0.017*
Posttreatment 3D ADC_kurtosis_	3.6 [3.0; 4.9]	5.1 [4.2; 5.5]	0.258
	n = 9	n = 2	
Percent change 3D ADC_mean_	10.6 [-2.4;20.4]	69.8 [28.1;111.5]	0.099
Percent change 3D ADC_skewness_	-67.5 [-81.7;-21.3]	-31.2 [-153.6;91.3]	1.000
Percent change 3D ADC_kurtosis_	-21.9 [-42.6;23.4]	-1.7 [-49.8;46.4]	0.637

3D ADC, apparent diffusion coefficient values derived from whole-tumor volume.

*indicates statistical significance.

### ROC analysis of treatment response

There was no statistical significance in AUC in the 5-level confidence scores of the standard MRI between the two readers (reader 1, 0.740, *P* = 0.157; reader 2, 0.606, *P* = 0.533). The ROC analysis of standard MRI for treatment response is summarized in Table 1.

**Fig 3:**
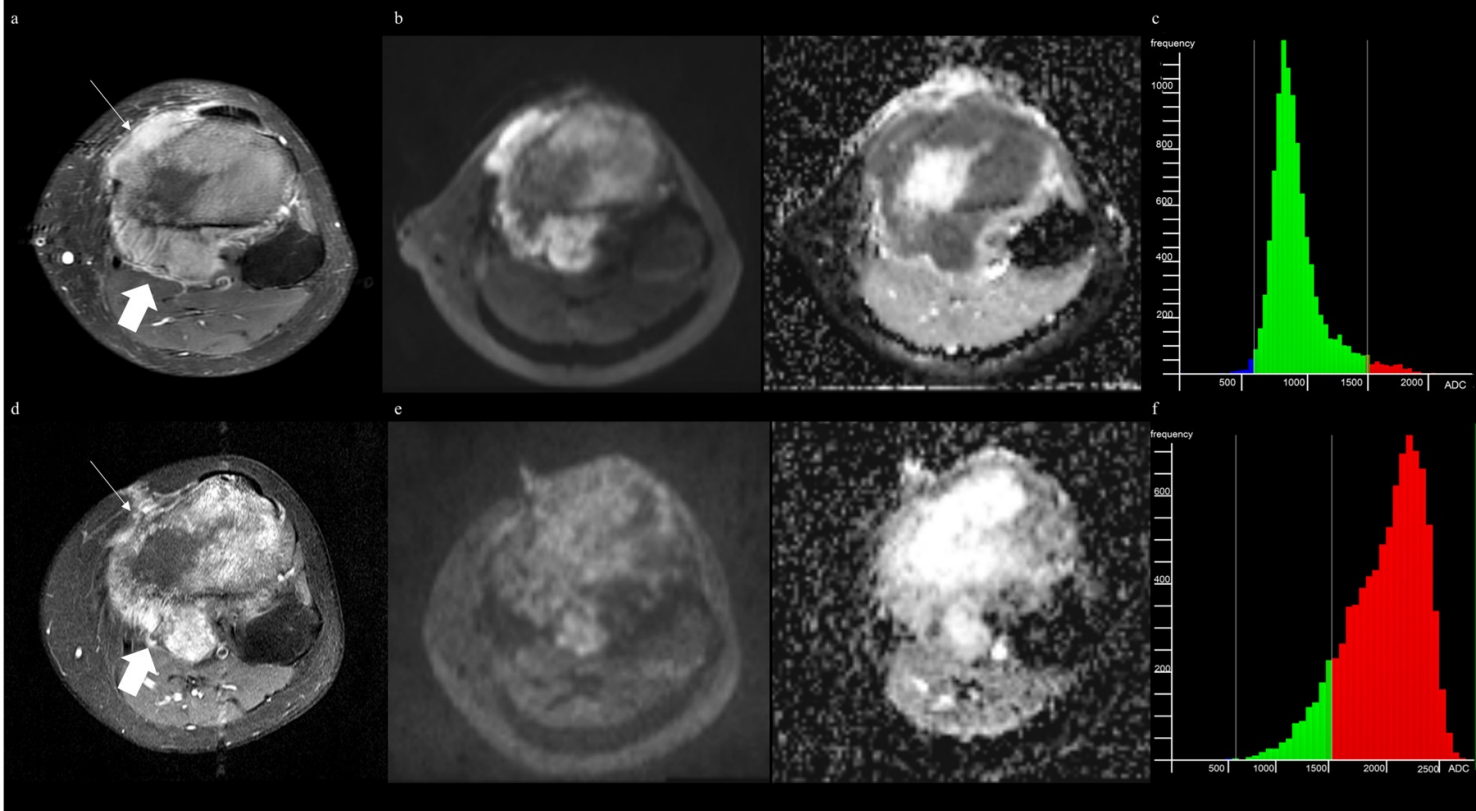
MRI of fibroblastic osteosarcoma and applying ADC values derived from single-section ROI can complement diagnostic ability. (A) Axial fat-suppressed (FS) contrast-enhanced T1-weighted image (T1WI) before treatment shows a tumor in proximal tibia with extraosseous lesions (arrows). (B) DWI (*b* of 800 sec/mm^2^) with ADC map before treatment shows 2D ADC_minimum_ and 2D ADC_mean_ of 870μm^2^/sec and 1011μm^2^/sec, respectively. (C) ADC histogram derived from whole-tumor volume before treatment shows 3D ADC_mean_ of 943μm^2^/sec, 3D ADC_skewness_ of 1.54, and 3D ADC_kurtosis_ of 6.83. (D) Axial FS contrast-enhanced T1WI after treatment shows the little change in size with heterogeneously enhancing extraosseous lesion (thick arrow), interpreted as equivocal in both readers. (E) DWI with ADC map after treatment shows 2D ADC_minimum_ and 2D ADC_mean_ of 1542μm^2^/sec and 2107μm^2^/sec, respectively, indicating a good responder. (F) ADC histogram derived from whole-tumor volume after treatment shows 3D ADC_mean_ of 1994μm^2^/sec, 3D ADC_skewness_ of -0.82, and 3D ADC_kurtosis_ of 3.43. The percent change of 2D ADC_minimum_ and 2D ADC_mean_ present as 77.2% and 105.6%, respectively. At histopathology, the tumor showed more than 95% necrosis, demonstrating a good responder.

**Fig 4:**
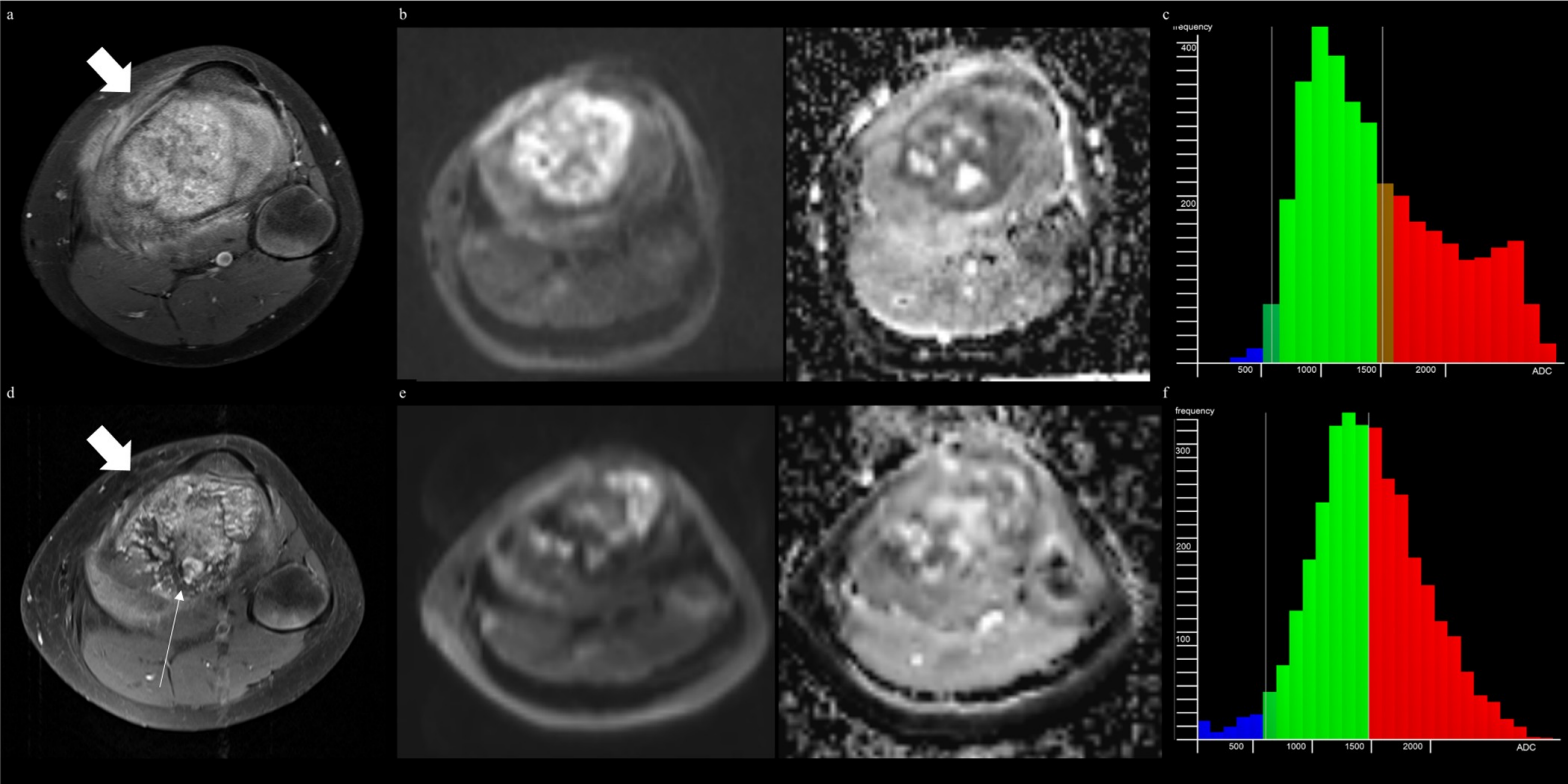
MRI of osteoblastic osteosarcoma and applying ADC values derived from single-section ROI can complement diagnostic ability. (A) Axial fat-suppressed (FS) contrast-enhanced T1-weighted image (T1WI) before treatment shows a tumor in proximal tibia with extraosseous lesion (thick arrow). (B) DWI (*b* of 800 sec/mm^2^) with ADC map before treatment shows 2D ADC_minimum_ and 2D ADC_mean_ of 880μm^2^/sec and 1179μm^2^/sec, respectively. (C) ADC histogram derived from whole-tumor volume before treatment shows 3D ADC_mean_ of 1472μm^2^/sec, 3D ADC_skewness_ of 0.55, and 3D ADC_kurtosis_ of 2.26. (D) Axial FS contrast-enhanced T1WI after treatment shows marked decrease in extraosseous lesion (thick arrow) with heterogeneously enhancement (thin arrow), interpreted as good responder in reader 2. (E) DWI with ADC map after treatment shows 2D ADC_minimum_ and 2D ADC_mean_ of 1047μm^2^/sec and 1395μm^2^/sec, respectively, indicating a poor responder. (F) ADC histogram derived from whole-tumor volume after treatment shows 3D ADC_mean_ of 1500μm^2^/sec, 3D ADC_skewness_ of 0.10, and 3D ADC_kurtosis_ of 3.15. The percent change of 2D ADC_minimum_ and 2D ADC_mean_ presents as 19.0% and 18.3%, respectively. The histopathology demonstrates a poor treatment response (necrosis = 32%).

Posttreatment and percent change of 2D ADC_minimum_ and 2D ADC_mean_ showed statistically significant AUC for reader 1, while the same parameters except percent change of 2D ADC_mean_ showed statistically significant AUC for reader 2 (*P* < 0.05) for discriminating between good and poor responders (Figs 3 and 4). The ROC analysis of ADC values derived from single-section ROI (2D ADC) with optimal cutoff values is summarized in Table 4.

**Table 4:** Diagnostic performances of 2D ADC measurement for treatment response.

Parameters	Cutoff	Sensitivity	Specificity	AUC
Pretreatment 2D ADC_minimum_				
Reader 1	≤956	78%	50%	0.639
Reader 2	>765	78%	50%	0.556
Pretreatment 2D ADC_mean_				
Reader 1	>1011	89%	50%	0.667
Reader 2	>1001	78%	50%	0.611
Posttreatment 2D ADC_minimum_				
Reader 1	≤1442	85%	100%	0.885*
Reader 2	≤1481	77%	75%	0.788*
Posttreatment 2D ADC_mean_				
Reader 1	≤2079	85%	75%	0.904*
Reader 2	≤1783	69%	75%	0.788*
Percent change 2D ADC_minimum_				
Reader 1	≤37.73	78%	100%	0.889*
Reader 2	≤39.3	100%	100%	1.000*
Percent change 2D ADC_mean_				
Reader 1	≤34.73	100%	100%	1.000*
Reader 2	≤40.51	100%	50%	0.778

2D ADC, apparent diffusion coefficient values derived from single-section regions of interest.

AUC, areas under the curve.

* indicates statistical significance.

**Fig 5:**
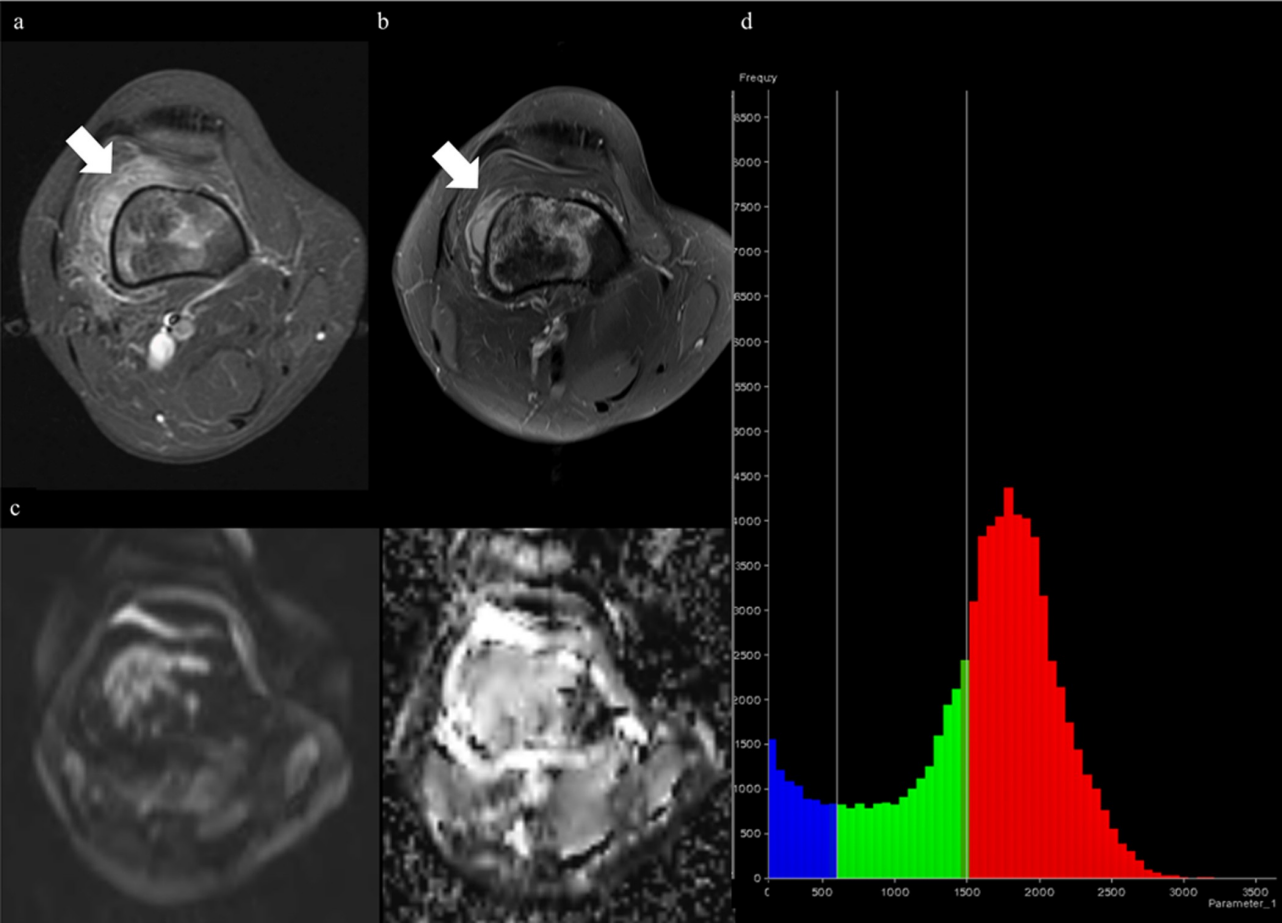
MRI of osteoblastic and chondroblastic osteosarcoma, lack of pretreatment DWI, indicating that ADC values derived from whole-tumor volume can reinforce diagnostic ability. (A) Axial fat-suppressed (FS) contrast-enhanced T1-weighted image (T1WI) before treatment shows a tumor with extraosseous lesion (thick arrow) in distal femur. (B) Axial FS contrast-enhanced T1WI after treatment shows slight decrease in extraosseous tumor size (thick arrow), interpreted as equivocal in reader 1. (C) DWI (*b* of 800 sec/mm^2^) with ADC map after treatment shows 2D ADC_minimum_ and 2D ADC_mean_ of 1235μm^2^/sec and 1968μm^2^/sec, respectively. Posttreatment values are also equivocal because of neighboring cutoff values and lack of pretreatment DWI. (D) ADC histogram derived from whole-tumor volume after treatment shows 3D ADC_mean_ of 1864μm^2^/sec, 3D ADC_skewness_ of 0.185, and 3D ADC_kurtosis_ of 4.14, suggesting a poor responder. The histopathologic finding demonstrates a poor treatment response (necrosis = 70%).

Posttreatment and percent change of 3D ADC_mean_ and posttreatment 3D ADC_skewness_ showed statistically significant AUC (*P* < 0.05) for treatment response (Fig 5). The ROC analysis of ADC values derived from whole-tumor volume with optimal cutoff values is summarized in Table 5.

**Table 5:** Diagnostic performances of 3D ADC measurement for treatment response.

Parameters	Cutoff	Sensitivity	Specificity	AUC
Pretreatment 3D ADC_mean_	>943.24	89%	50%	0.667
Pretreatment 3D ADC_skewness_	≤1.45	89%	50%	0.556
Pretreatment 3D ADC_kurtosis_	≤6.33	89%	50%	0.611
Posttreatment 3D ADC_mean_	≤2039.17	85%	50%	0.846*
Posttreatment 3D ADC_skewness_	>-0.82	85%	100%	0.904*
Posttreatment 3D ADC_kurtosis_	≤4.9	77%	75%	0.692
Percent change 3D ADC_mean_	≤45.1	89%	50%	0.889*
Percent change 3D ADC_skewness_	>-153.65	100%	50%	0.500
Percent change 3D ADC_kurtosis_	≤39.43	100%	50%	0.611

3D ADC, apparent diffusion coefficient values derived from whole-tumor volume.

AUC, areas under the curve.

*indicates statistical significance.

### Multivariate logistic regression analysis for predicting poor responders

Based on the stepwise multivariate logistic regression analysis, the best predictors for poor responders were posttreatment 2D ADC_mean_ (odds ratio, 0.994; 95% confidence interval, 0.986–1.002]) of reader 1 and none of reader 2 among ADC values obtained from the single-section ROI and posttreatment 3D ADC_skewness_ (odds ratio, 62.08; 95% confidence interval, 0.62–6221.71]) among ADC values obtained from the whole-tumor volume.

**Fig 6:**
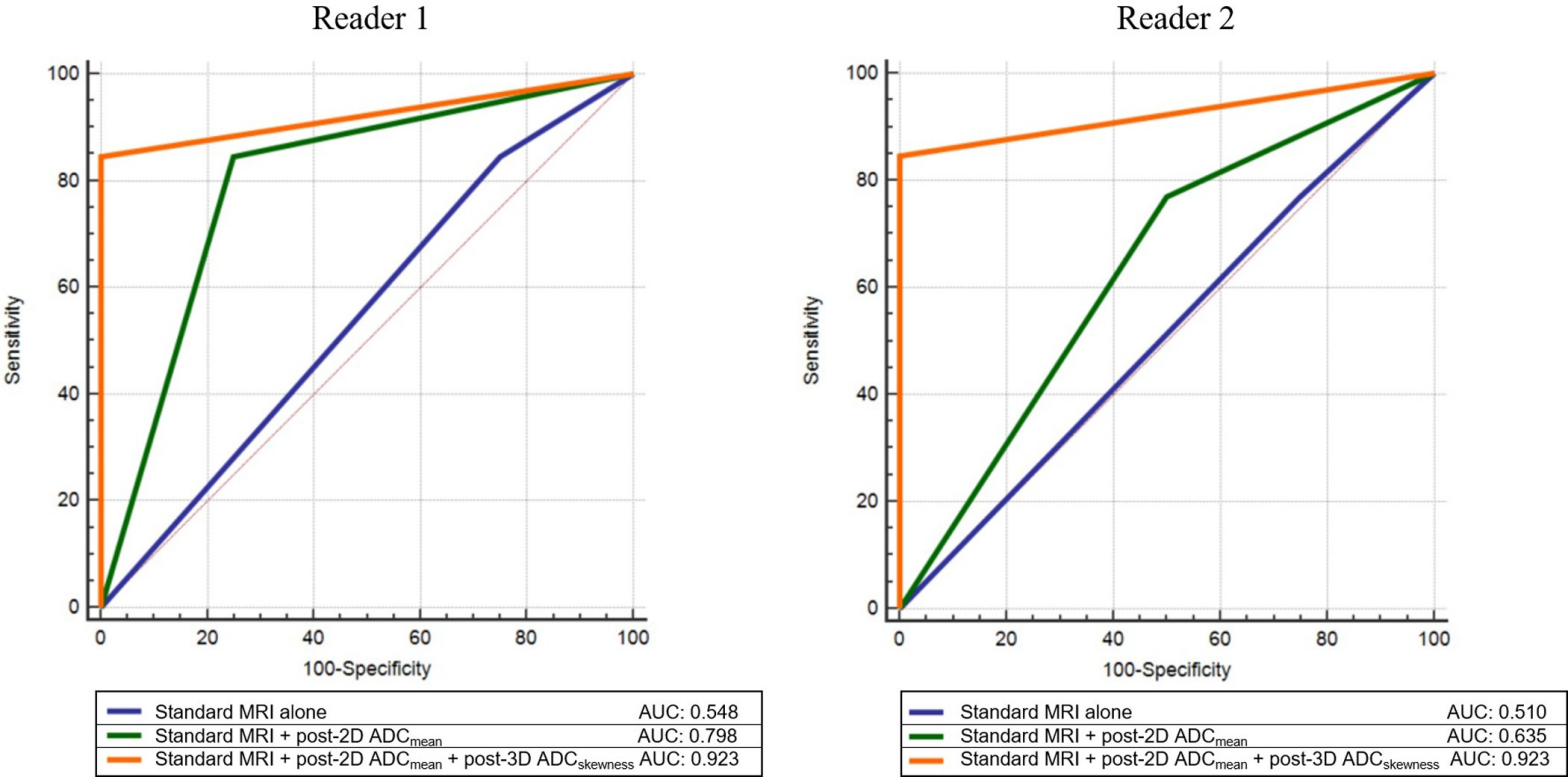
ROC comparison between three prediction models for both readers. AUC is increased by adding parameters to standard MRI. The 1^st^ model: standard MRI alone, the 2^nd^ model: standard MRI with posttreatment 2D ADC_mean_, the 3^rd^ model: standard MRI with posttreatment 2D ADC_mean_ and posttreatment 3D ADC_skewness_. post- = posttreatment; 2D = single-section ROI; 3D = whole-tumor volume; ROC = receiver operating characteristic; AUC = areas under the curve.

Three prediction models were designed as follows: 1^st^ model, standard MRI alone; 2^nd^ model, standard MRI combined with posttreatment 2D ADC_mean_; and 3^rd^ model, standard MRI combined with posttreatment 2D ADC_mean_ and posttreatment 3D ADC_skewness_. Each of the models showed sensitivity and specificity as follows: 85% and 25%; 85% and 75%; and 85% and 100% for reader 1 and 77% and 25%; 77% and 50%; and 85% and 100% for reader 2, respectively. Each of the models showed the following AUC values: 0.548, 0.798, and 0.923 for reader 1; and 0.510, 0.635, and 0.923 for reader 2, respectively (Fig 6). Other model of standard MRI combined with posttreatment 3D ADC_skewness_ also showed sensitivity and specificity of 85% and 100% with AUC of 0.923, same as 3^rd^ model.

## Discussion

Our study showed that the addition of DWI including a volumetric analysis to standard MRI improved the diagnostic accuracy for determining poor responders to neoadjuvant chemotherapy among osteosarcoma patients. Among the parameters obtained from single-section ROI, posttreatment mean ADC was the best independent predictor for poor responder. On the other hand, posttreatment skewness of ADC obtained from whole-tumor volume in addition to posttreatment mean ADC obtained from single-section ROI were helpful for less experienced readers.

Osteosarcoma is the most common type of malignant bone tumor with a peak incidence in the second decade of life [28]. It arises within bone and may metastasize to lung [19]. A combination of surgery and chemotherapy is the choice of treatment, which improved the survival rates [29]. However, there are still 20 ~ 30% of patients with poor curative effect of limb salvage surgery, and the extent of tumor necrosis to neoadjuvant chemotherapy has been known to be the most important prognostic factor in patients with localized disease [20]. Traditionally, the therapeutic effectiveness of chemotherapy was assessed by comparison of tumor size before and after therapeutic intervention [30]. However, for the osteosarcomas, there was a specific issue; the tumor size showed little changes after neoadjuvant chemotherapy [12, 31], despite successful chemotherapy. The reason was that the chemotherapy on osteosarcomas has only affected on the mineralized matrix of tumor [10]. According to Lang et al. [22], signal intensity (SI) changes on T2WI are sometimes nonspecific because both viable and necrotic tissues can demonstrate similar SI. The main reason for misinterpretation based on standard MRI could be related to the granulation tissue or fibrosis being interpreted as viable enhancing solid portions [12, 31, 32]. If the treatment response to neoadjuvant chemotherapy cannot be accurately evaluated, it will have an adverse effect to surgical planning, adjuvant chemotherapy selection, and prognostic judgement [20]. Therefore, it is necessary to find an effective and quantitative method to evaluate the treatment response.

DWI may help differentiate granulation/fibrotic tissue from viable tumors [10, 14, 16, 22]. In previous studies, the treatment response of osteosarcoma was assessed with DWI using single-section ROI (2D ADC) on a representative axial image [6, 10, 14, 16, 20, 22]. ADC measurement reduces the number of misleading cases by using parameters including percent changes of 2D ADC and posttreatment 2D ADC values. Many previous studies have reported that ADC difference and ADC ratio were greater in good responders than in poor responders [6, 10, 14, 15]. One study reported that the ADC_mean_ showed a significant correlation with treatment response as the best predictor of treatment [17]. However, another study showed that the significant difference between good and poor responders was not in ADC_mean_ ratio; rather, it was in ADC_minimum_ ratio [16]. ADC_minimum_ ratio well reflects not only the highest cellular portions but also the treatment response in a similar context of SUV_max_, which represents the point of highest metabolic activity in a tumor [8, 33]. This inconsistency may be attributed to differences in experience and interpretation, ROI methods, MRI vendors, and MRI parameters among readers and studies for reflecting whole-tumor heterogeneity from single-section analysis. This inconsistency could also be due to reader experience since assessments using ADC with a single-section ROI may have low reproducibility in less experienced readers [18]. Furthermore, DWI interpretation of poor responders with extraosseous myxoid component or with the chondroblastic osteosarcoma subtype, in which ADC values were similar to those of tumor necrosis [34]. Therefore, we thought that ADC_mean_ could better reflect the tumor heterogeneity than ADC_minimum_ value and found that ADC_mean_ was the best independent predictor for poor responders among the parameters obtained from single-section ROI.

Whole-tumor volume analysis of the ADC map may complement these limitations of single-section ROI measurement [18]. One study reported that ADC_mean_ ratio, skewness, and kurtosis derived from whole-tumor volume were well correlated with the therapy-induced response [19]. Another report demonstrated that posttreatment ADC_mean_ derived from whole-tumor volume in good responders was higher than that of poor responders [20]. In our study, posttreatment 3D ADC_skewness_ derived from whole-tumor volume analysis of the ADC histogram was helpful for predicting poor responders, especially less experienced readers or patients with no available pretreatment DWI or the chondroblastic osteosarcoma subtype. Like our results, Wang et al [20] reported significant differences in ADC_mean_ and peak of the ADC histogram after neoadjuvant chemotherapy between good and poor responders. However, Wang et al [20] analyzed ADC histograms visually and did not use quantitative measurements such as ADC_skewness_ or ADC_kurtosis_. Based on our study findings, quantitative ADC histogram analysis derived from whole-tumor volume may allow easy and quick perception of treatment response because a negative skewness of ADC value derived from whole-tumor volume after chemotherapy is related to a higher proportion of tumor necrosis in good responders, causing ADC histograms to have a right-sided peak.

We demonstrated the feasibility of posttreatment DWI for assessing treatment response. A similar result that post-neoadjuvant chemotherapy ADC value in good responders was significantly higher than that of poor responders was noted in one study of osteosarcoma [20]. These results suggested that treatment efficacy could be evaluated without comparison of the initial examination.

There were several limitations to our study. First, it was a retrospective study and, therefore, subject to selection bias. Second, a small number of patients from a single institution was included. Third, pretreatment DWI was not available for 6 of the 17 patients; thus, the evaluation using percent change was limited. Fourth, we used only two common *b* values of 0 and 800 sec/mm^2^ because protocols have changed in our institution. And finally, histopathological whole-tumor mapping of specimens was not performed as in other studies.

In conclusion, the addition of DWI including a volumetric analysis to standard MRI may improve the diagnostic performance of predicting poor responders to neoadjuvant chemotherapy in patients with in osteosarcoma at 3T. Posttreatment mean ADC obtained from single-section ROI and posttreatment skewness of ADC obtained from whole-tumor volume may be the best predictors for poor responders in patients with osteosarcoma.

## Supporting information

S1 Dataset (XLSX)
